# Supplementary material for: Engineering Terpene Production Pathways in Methylobacterium extorquens AM1
Source: Microorganisms. 2024 Feb 29;12(3):500. doi: 10.3390/microorganisms12030500 (PMC10974752; doi:10.3390/microorganisms12030500)
Supplement: Supplementary file 1 [file microorganisms-12-00500-s001.zip › microorganisms-2863394-supplementary.pdf]

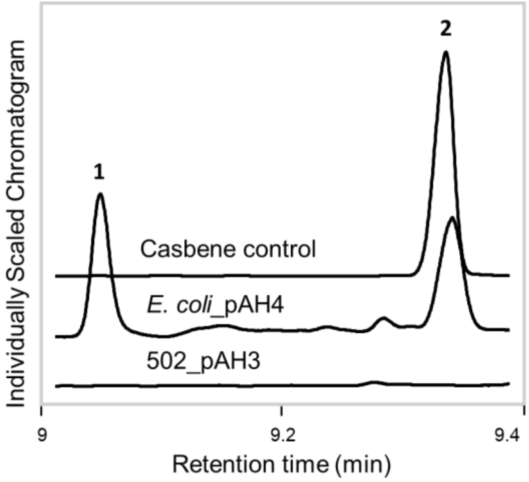

Supplemental Figure S1: GC-FID confirmation of casbene production. Extracts from *E. coli* and *M. extorquens* casbene sample chromatograms are compared to a positive sample. The *E. coli* samples from the constitutive promoter when compared to a casbene standard (2) show success of the system at retention time of 9.35 minutes. (1), Geranylgeraniol, product of unspecific phosphatase activity from geranylgeranyl diphosphate.
